# Health Tract, No. 274

**Published:** 1867-02

**Authors:** 


### BODILY STRENGTH.

Every grain of bodily power, ability of motion, or effort, is derived from the food we eat; stimulants, spirits, and mental excitement call out that power in unnatural quantities, but the power itself is derived from the food, through the agency, the medium of the nerves and muscles. The food does three things before power is produced. It warms, it builds up, it repairs; and ability to work is the result. This process of repair and building may be called nourishing. In general, the elements of all power proper for animal life contain both warmth and nourishment, such as meat and bread: but fruits impart no warmth; while oils, sugar, and starch contain little else than the articles of warmth.

The first necessity for infants is warmth; the milk on which they feed contains both an oil and a sugar. They would die without sweets. The body is warmed by process of combustion, a burning up of the carbon of the system. Sugars, oils, and starches, are almost entirely carbon. This burning of carbon is kindled and carried on by means of the air which is breathed into the lungs.

The more we exercise, the faster or deeper we breathe, and the more air we take into the lungs; hence exercise is said to "warm us up." The product of combustion is carbonic acid, which is exhaled from the lungs The amount of this acid exhaled during sleep is represented by nineteen; while lying down awake, twenty-three; while walking at the rate of two miles per hour, seventy-three; three miles per hour, one hundred and seventy-five.

Now, as the quantity of carbonic acid gas passing out of the system represents the amount of impurieties carried from it, it is seen at once, the powerful agency which exercise has in rendering the blood pure, and keeping it so; for this carbonic acid is taken out of the blood in the lungs by the process of breathing.

When sitting in a room, this carbonic acid gas which comes from the lungs, hovers around the head and face to some extent, and is, with all its impurities, rebreathed; but when working, riding, or walking out of doors, it is carried immediately away by the wind; hence, every breath of out-door air is a pure breath; hence, too, the great superiority of out-door exercise as a means of health, above indoor quiet.

Different kinds of food have various proportions of the elements of power; that is, we can work longer on a pound of bread than on a pound of cabbage.

Scientific men have arrived at the following proportions. The table should be read thus: One pound of cabbage gives two degrees of strength,

| | | |
|---|---|---|
| Milk 2, | Lean Beef, 6, | Flour, 18, |
| Cabbage, 2, | Mackerel, 7 1-2, | Lump Sugar, 18, |
| Carrots, 2 1-2, | Hard boiled egg, 10, | Cheshire cheese, 19 |
| White of egg, 2 1-2, | Bread, 12 | Arrowroot, 19, |
| Apples, 3, | Isinglass, 17, | Cocoa Nibs, 32, |
| Butter, 3 1-2, | Rice, 17 1-2. | Fat Beef, 41, |
| Potatoes, 5, | Oatmeal, 18, | Cod Liver Oil, 41. |

